# In memoriam – Dr. Mahabir Gupta 1942–2020

**DOI:** 10.1080/13880209.2020.1871032

**Published:** 2021-02-03

**Authors:** Valdir Cechinel-Filho

**Affiliations:** Nucleo Invest Quimicofarmaceut, Univ Vale Itajai, Itajai, Brazil


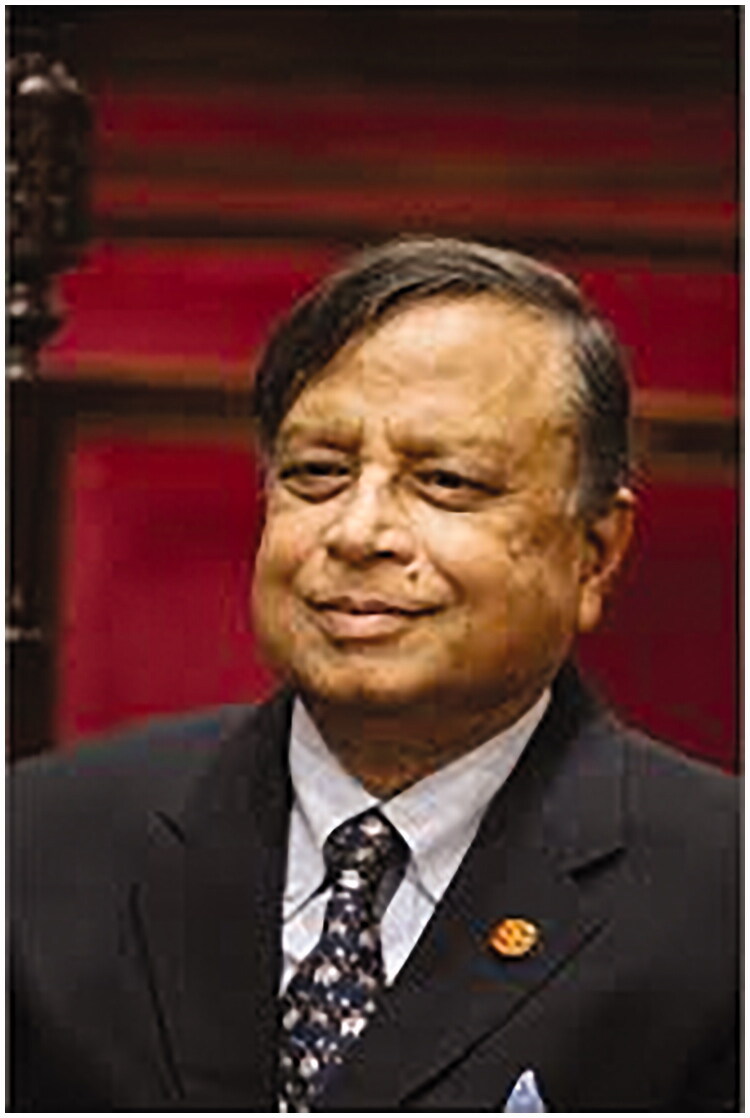


Mahabir Gupta was born in India in 1942. He received his Bachelor and Master's degrees in Pharmacy from the University of Rajasthan and Banaras Hindu University, respectively, and earned his Ph.D. at Washington State University, Pullman, WA, USA.

He joined the College of Pharmacy of the University of Panama in 1972, dedicating his highly productive professional life to pharmacognosy research in Panama and several other countries, especially Latin America. Over the years, in additional to his professorial position at the University of Panama, he served as Scientific Advisor to the Vice-President for Research and Graduate Studies, Director of International Technical Cooperation, Head of the Department of Medicinal Chemistry & Pharmacognosy, Advisor to the President of University of Panama International Technical Cooperation, and Dean, College of Pharmacy.

In addition to the above, Dr. Gupta was an International Coordinator of the Natural Products Drug Discovery Program of the Ibero-American Program of Science and Technology for Development (CYTED) during 1995–2005, and Coordinator, Area of Health, CYTED 2011–2014, for 19 countries of Latin America, Spain and Portugal, with a network of over 1500 scientists. He was the Executive Director of the INTERCIENCIA Association for the promotion of science in the Americas.

At the time of his death, he was Emeritus Professor of Pharmacognosy and Founding Director of the Centre for Pharmacognostic Research on Panamanian Flora at the University of Panama, Panama City, Panama, and Manager, Health Area, Iberoamerican Program of Science and Technology for Development CYTED, Madrid.

In recognition of his outstanding performance and training of researchers, Dr. Gupta received many awards and honours. Examples include the National Research Achievement Award, National Secretary of Science Technology, and Innovation Panama; 2010 Science Award, Panamanian Association for the Advancement of Science; Distinguished Scientist − 2008 in the National Investigators System National Secretariat for Science Technology & Innovation of Panama; Interciencia Award 2008 for Health Sciences; Honorary Professor National University of San Marcos, Perú; Vice Chairman, Plant Sciences Group, International Organisation of Chemical Sciences for Development (IOCD), since 2000; Member Dietary Supplements Botanical Expert Committee, U. S. Pharmacopoeia, USA 2005–2010; Member Medicinal Plants Expert Group, International Centre for Science and High Technology (ICS/UNIDO), Trieste, Italy; Recipient of International Scientific Cooperation Award from the American Association for the Advancement of Science; Member Board of Directors of International Foundation for Science; Member Board of Directors of the International Council of Medicinal and Aromatic Plants; Member of the International Union for Conservation of Nature and Natural Resources/Species Survival Commission, Ethnobotany Specialist Group; Member, ICSU Regional Committee for Latin America and the Caribbean; Academy of Sciences of Latin America, Caracas, Venezuela; Royal Academy of Pharmacy, Madrid, Spain; Panamanian Academy of Medicine and Surgery; Panamanian Association for the Advancement of Science – Founding Member and Permanent Secretary (Represents in IANAS because of lack of a science academy in Panama).

Further, Dr. Gupta was elected Twas Fellow, TWAS World Academy of Sciences for the Advancement of Science in Developing Countries, and he received the maximum honour (Doctor *Honoris causa*) from the Universidad Nacional Autónoma de Amazonia Peruana, Iquitos, Perú in 2005, and the University of Vale do Itajaí (UNIVALI Itajaí-Brazil) in 2014.

Reflective of his global presence, Dr. Gupta visited over 50 countries, and present 240 invited lectures. He was fluent in English, Spanish and Hindi, and had a good reading knowledge of German. He will be remembered always for his indelible scientific contributions, with the publication of several books and about 300 manuscripts on medicinal plants and drug discovery. Dr. Gupta was an active Associated Editor of *Pharmaceutical Biology* and member of Editorial Boards of many respected journals.

Those who had the privilege of knowing Mahabir Gupta will remember his passion for science, warm and caring heart, welcoming spirit, entrepreneurship, and uncanny ability to promote collegiality. He leaves his wife, Olga Gupta, and their three children, Rajesh, Sanjay and Purni, in addition to thousands of friends and admirers, on five continents.

## Notice from the editor and managing editor

We deeply regret the untimely loss of our dear friend and colleague, Mahabir Gupta. As a gesture of our deep respect and admiration, and as a token of thanks for his tireless efforts that vastly improved the journal, issue 58-01 (2020) of *Pharmaceutical Biology* is dedicated to his memory.

